# Concurrent presentation of thyroid storm and diabetic ketoacidosis: a systematic review of previously reported cases

**DOI:** 10.1186/s12902-019-0374-3

**Published:** 2019-05-17

**Authors:** Devarajan Rathish, Senuri Karalliyadda

**Affiliations:** Department of Pharmacology, Faculty of Medicine and Allied Sciences, Rajarata University of Sri Lanka, Saliyapura, 50008 Sri Lanka

**Keywords:** Thyrotoxic crisis, Thyroid crisis, Diabetes mellitus, Anti-thyroid drugs, Anti-diabetic drugs, Case reports

## Abstract

**Background:**

Simultaneous development of thyroid storm and diabetic ketoacidosis (DKA) is a rare condition. The review aims to summarise its clinical presentation, investigation findings and treatment options.

**Methods:**

Databases and reference lists of the selected articles were searched for case reports in English which describe concurrent presentation of thyroid storm and diabetic ketoacidosis. CARE guidelines were used for the quality assessment of the selected articles.

**Results:**

Twenty-six cases from twenty-one articles were selected out of 198 search results. Western Pacific, and American regions contributed to 77% of the cases. Females were most affected (88%). Features of Graves’ disease like hyperthermia and tachycardia, gastrointestinal and neuro-psychiatric disturbances were the common clinical presentations. In most of the cases, previous diagnosis of diabetes mellitus preceded that of Graves’ disease (46%). Among patients having their drug compliance reported, all had poor compliance to their routine anti-thyroid (9/9) and anti-diabetic (2/2) agents. Moreover, in all cases where HbA_1C_ (7/7) and T4 (16/16) were measured, the results were elevated and where TSH (17/17) was measured, the results were low. The recommended treatment for DKA and thyroid storm was used in most cases and methimazole was the thionamide of choice in the latest four cases reported. All cases survived except four (15%).

**Conclusions:**

Concurrent presentation of thyroid storm and diabetic ketoacidosis is rare but life-threatening. Therefore, efforts should be made to maximise patient compliance to anti-thyroid and anti-diabetic agents in treating such patients.

**Electronic supplementary material:**

The online version of this article (10.1186/s12902-019-0374-3) contains supplementary material, which is available to authorized users.

## Background

Diabetic ketoacidosis (DKA) is a serious metabolic state due to lack of insulin, and it is usually seen among patients with type 1 diabetes mellitus [1]. DKA is characterised by hyperglycaemia, ketosis and metabolic acidosis [2]. Females had higher prevalence of DKA [3]. Although, age 18 to 44 years is more prone for DKA, the extremes of ages show higher mortality in DKA [1]. The most common precipitating factors for DKA in the developed countries were poor adherence to insulin therapy, infection and newly diagnosed diabetes mellitus. However, infections and poor access to care were the most common precipitating factors in developing countries [4]. A systematic review reported a prevalence of 0–128 per 1000 people and an incidence of 0–56 per 1000 person-years for DKA [3].

Thyroid storm (TS) or thyrotoxic crisis or thyroid crisis is “the extreme manifestation of thyrotoxicosis, presenting as a clinical syndrome of multi-organ dysfunction, with or without a precipitating cause” [5]. It often follows sudden cessation of antithyroid drugs, surgeries, trauma, acute illnesses, drug reactions, parturition, use of iodinated contrast medium and radioiodine therapy but, it can also occur without an apparent cause [6]. Females are more prone to develop TS, and all age groups are affected [7]. TS related mortality was found to be associated with shock, intravascular coagulation and multi-organ failure [8]. The incidence of thyroid storm was reported as 0.2 per 100,000 population per year from Japan [9].

Potenza M and colleagues (2009) stated that the variations in “insulin secretion, insulin clearance, gluconeogenesis, glycogen synthesis, glucose oxidation, nonoxidative glucose metabolism, adipokine signalling, and lipid oxidation” would increase blood glucose levels and insulin resistance among patients with excessive thyroid hormone [10]. The above mechanism could disturb the patient’s diabetic control and lead to DKA which in-turn would cause thyroid storm [10]. We aim to systematically review cases reported on concurrent presentation of TS and DKA concerning its presentation, investigations and management. In 2009 (9 cases) and 2017 (18 cases), two separate summaries on concurrent presentation of TS and DKA were reported along with similar case reports [10, 11]. However, this systemic review includes additional cases and an in-depth analysis.

## Methods

### Eligibility criteria

The review included all case reports published on concurrent presentation of TS and DKA. The Burch-Wartofsky Point Scale (BWPS) [12] and the Japan Thyroid Association criteria (JTA) [13] were used to define TS and its severity. The severity of DKA was defined using the diagnostic criteria for diabetic ketoacidosis [1]. In cases where an arterial blood gas analysis on admission was not reported, the diagnosis of DKA by the relevant authors of the case report was considered. Reports in non-English language were excluded. Reports were not excluded based on the year of publication or patient population.

### Information sources and search strategy

The research was done in September 2018. Electronic databases and grey literature were searched using a string of keywords (Table 1). PubMed (Advanced search) [14], Science Direct (Advanced search) [15], Trip (Search) [16], The Cochrane Library (Advanced search) [17], Google Scholar (Advanced search) [18] and Google search (verbatim) [18] were searched. Further, the reference lists of the selected reports were searched for relevant cases. MeSH and other related terms were used to obtain optimum data.

**Table 1:** Keywords for databases and the number of search results

Database and Keywords	No of search results
*PubMed (Advanced search)* (((((((“thyroid storm”[Title/Abstract]) OR Thyroid storm[MeSH Terms]) OR “thyroid crisis”[Title/Abstract]) OR “thyroid crisis”[MeSH Terms]) OR “thyrotoxic crisis”[Title/Abstract]) OR thyrotoxic crisis[MeSH Terms])) AND ((((“diabetic ketoacidosis”[Title/Abstract]) OR “diabetic ketoacidosis”[MeSH Terms]) OR “ketoacidosis”[Title/Abstract]) OR ketoacidosis[MeSH Terms])	41
*Science Direct (Advanced search)* (Thyroid storm OR thyroid crisis OR thyrotoxic crisis) AND (Diabetic ketoacidosis OR ketoacidosis)	9
*Trip (Search)* (Thyroid storm OR thyroid crisis OR thyrotoxic crisis)(Diabetic ketoacidosis OR ketoacidosis)	23
*The Cochrane Library (Advanced search)* Thyroid storm OR thyroid crisis OR thyrotoxic crisis (Title Abstract Keyword) AND Diabetic ketoacidosis OR ketoacidosis (Title Abstract Keyword)	1
*Google Scholar (Advanced search)* allintitle: (Thyroid storm OR thyroid crisis OR thyrotoxic crisis) AND (Diabetic ketoacidosis OR ketoacidosis)	1
*Google Search (Verbatim)* (Thyroid storm OR thyroid crisis OR thyrotoxic crisis) AND (Diabetic ketoacidosis OR ketoacidosis)	123
Total	198

### Study selection

Both DR and SK were involved in study selection. DR performed a comprehensive literature search. SK independently screened the titles and abstracts of all identified studies for selection, according to the inclusion criteria. The selected study was independently reviewed by DR to confirm the eligibility.

### Data collection process, data items and data analysis

Demographic data, clinical presentation, investigation findings and the management plan were extracted from the selected studies. The units of measurements were presented in SI units. The data were analysed using Microsoft Excel (Additional file 1). Descriptive statistics were used to describe the data. The quality of the case reports was assessed using the CARE guidelines [19]. A score of one was given for each item outlined in the CARE guidelines with a maximum score of 30 for a case report. This systematic review was reported according to the Preferred Reporting Items for Systematic survey and Meta-Analysis (PRISMA) statement [20] (Additional file 2).

## Results

### Selected case reports

**Fig. 1:**
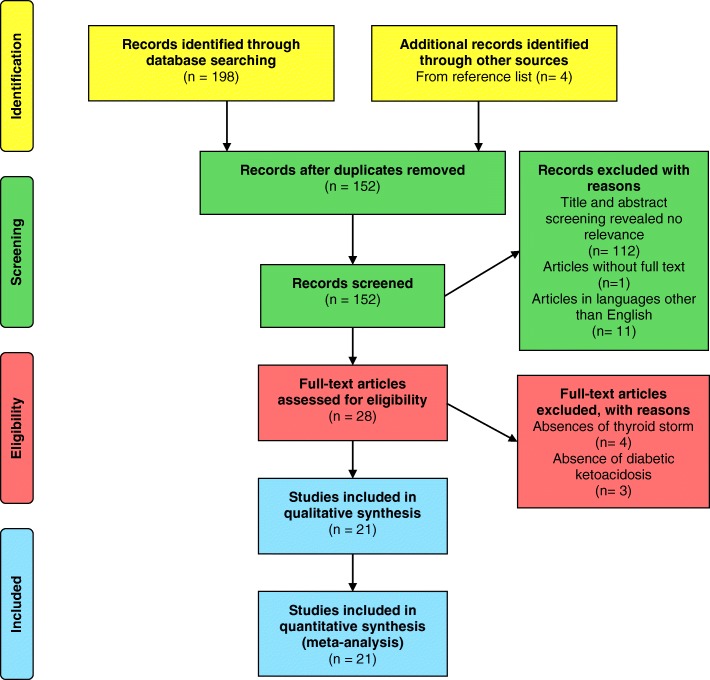
Flow diagram showing the selection process of articles for this review, according to PRISMA 2009

A total of 198 results were found from databases (Table 1), and 4 reports were selected from the reference lists. Following the exclusion of duplicates, 152 articles underwent title and abstract screening out of which 112 articles were excluded due to irrelevance to the review objective. One report was excluded due to unavailability of full-text [21]. Another 11 reports were excluded as these were in Swedish [22], Serbian [23], Russian [24, 25], Japanese [26, 27] and German [28–32] languages. The full-texts of the remaining 28 reports were studied, and 7 were excluded due to mismatch with the selection criteria (absence of thyroid storm − 4, absence of DKA - 3). Finally, 21 reports were subsequently selected which had 26 cases [10, 11, 33–51] (Fig. 1). The mean score for the quality assessment of the selected 26 cases was 19.2 (SD ± 3.6). The minimum and the maximum score achieved were 14 and 25, respectively. Summary of scores for each item of the CARE checklist is given in Additional file 3.

### Characteristics of the selected studies

Additional file 1 contains extracted data from the 26 cases selected for this review. Eleven cases were reported from Western Pacific region (42%), nine from the region of Americas (35%), three each from European and Eastern Mediterranean regions (each 12%). There were no cases reported from the African or the South-East Asian region. The highest number of cases were reported from the United States of America (*n* = 11), followed by Taiwan (*n* = 4), Japan (*n* = 3), United Kingdom (*n* = 3), Saudi Arabia (*n* = 2), Republic of Korea (*n* = 1), New Zealand (*n* = 1) and Spain (*n* = 1). The first case study was found to be reported by Hanscom and Ryan in 1957 from the United States of America [33]. The latest case was reported by Cheng ET at el. in 2017 from Taiwan [49]. The mean age of the patients was 39.69 years (SD 19.94), with an age range of 16–81 years. There were 23 females (88%) and 3 males (12%). Two cases were less than 18 years and were from the Republic of Korea [39] and Taiwan [49]. The nationality of the patients was reported in only two cases. Those were Afghanis and Chinese from the Saudi Arabia [47] and New Zealand [36] respectively.

### Clinical presentations

More than half of the patients presented with either gastrointestinal (81%) or neurological symptoms (54%). Also, general features like fever, heat intolerance, lethargy, sweating, weakness and weight loss were seen in 73% of the cases. Other presenting complaints are shown in Table 2.

**Table 2:** Presenting complaints of the patients with both thyroid storm and diabetic ketoacidosis

Presenting complaint according to organ systems	Frequency out of 26 (%)
Gastrointestinal (abdominal fullness, abdominal pain, anorexia, diarrhoea, epigastric pain, nausea, polyphagia, vomiting)	21 (81)
General (fever, heat intolerance, lethargy, sweating, weakness, weight loss)	19 (73)
Neurological/ psychological (altered mental status, anxiety, confusion, delirium, distressed, dizziness, irritability, slurred speech, unconscious)	14 (54)
Cardiovascular (palpitation)	10 (39)
Dehydration (dry mouth, polydipsia)	10 (39)
Renal (polyuria)	9 (35)
Respiratory (chest pain, cough, dyspnoea, sore throat)	9 (35)
Musculoskeletal (back pain, inter-scapular pain, shoulder pain)	2 (8)

### Summary of results of Burch-Wartofsky point scale (BWPS) for the diagnosis of thyroid storm

All cases had a documented initial heart rate of > 100 beats per minute, and 73% (19/26) of the cases had an initial temperature of > 37.5 °C. More than half of the cases had central nervous system disturbance (58% - 15/26), gastrointestinal-hepatic dysfunction (92% - 24/26) and a probable precipitating event (54% - 14/26). Out of those cases reporting of central nervous system dysfunction, mild, moderate and severe manifestations were seen in 47% (7/15), 40% (6/15) and 13% (2/15) cases respectively. Out of those who had gastrointestinal-hepatic dysfunction, the majority had moderate manifestation (96% - 23/24) and one patient had severe manifestation. Atrial fibrillation and heart failure were reported in 6 and 2 cases respectively. Poor compliance with the anti-thyroid drug was the most common precipitating event (64% - 9/14). Influenza A (*n* = 2), under treated Grave’s disease (GD) (*n* = 2), automobile accident (*n* = 1), dilation and curettage (*n* = 1), post-surgery (*n* = 1), pregnancy and prepartum hypothyroidism treated with desiccated thyroid (*n* = 1) and septicaemia (*n* = 1) were the other possible precipitating events for TS. The mean (SD) score for BWPS was 62.3 (10.0) with a range of 40 to 80. The reported score and the reviewers’ score for BWPS did not tally in two case reports [39, 51]. One received extra 10 points for a probable precipitating event (poor drug compliance) [51] while the other did not report the breakdown of the scores [39].

### Summary of results of Japan thyroid association criteria for the diagnosis of thyroid storm

In all cases where T4 (16/16) were measured, the results were elevated whereas, 83% (5/6) of T3 levels were elevated. In all cases where TSH (17/17) was measured, the results were low. Most of the cases were graded as TS1 first combination (46% - 12/26), while 39% (10/26) were TS2 alternative combination, 12% (3/26) were TS2 first combination and the grading was unable to be performed in one case report.

### Summary of results of the diagnostic criteria for diabetic ketoacidosis

The mean serum bicarbonate and the anion gap fulfilled the “severe” category of DKA (Table 3). The mean arterial pH fulfilled the “moderate” category of DKA. Out of the cases reporting serum (*n* = 8) and urine (*n* = 14) ketones, all were found to be positive (100%). Out of the cases reporting the mental status, drowsiness was seen in 69% (9/13) while 15% (2/13) of the cases were alert and 8% (1/13) each had stupor and coma. Majority of the cases were having severe DKA (58% - 15/26), followed by moderate DKA in 27% (7/26), mild DKA in 12% (3/26) and the severity was unable to be assessed in one patient. Moreover, HbA_1C_ was reported in seven patients and the mean (SD) HbA_1C_ was 11.7 (2.4) % with a range of 7.7 to 14.6%. Details of other investigations including antibody levels are summarized in Additional file 1.

**Table 3:** Summary of laboratory results related to diagnostic criteria for diabetic ketoacidosis [1]

Name of investigation (unit)	Cases reported out of 26 (%)	Range	Mean (SD)
Anion gap (mmol/L)	6 (23)	17.0–36.0	27.8 (7.0)
Arterial pH	16 (62)	6.9–7.3	7.2 (0.1)
Effective serum osmolality (mmol/kg)	6 (23)	282.2–339.0	305.7 (21.7)
Plasma glucose (mmol/L)	24 (92)	18.7–48.5	29.8 (9.0)
Serum bicarbonate (mmol/L)	17 (65)	2.8–17.0	7.4 (4.0)

### Past medical history

Past history of DKA was reported in two cases from Japan [34] and Spain [41]. Diabetes mellitus (58% - 15/26), GD (35% - 9/26) and hypertension (8% - 2/26) were the three most common past medical illnesses (Additional file 1). Five patients were reported to have a past history of both diabetes mellitus and GD. Diabetes mellitus was diagnosed before GD in 12 cases (46%), GD before diabetes mellitus in 6 cases (23%) and the order of diagnosis was unable to be identified in 8 cases (31%) (Table 4). Patients were on Lugol solution, methimazole and propylthiouracil for the treatment of previously diagnosed GD. Compliance with anti-thyroid drugs was reported in 9 cases and all were reported to have poor compliance (Table 4). Patients were on insulin, metformin, sulfonylurea and thiazolidinedione for the treatment of previously diagnosed diabetes mellitus. Compliance with anti-diabetic drugs was reported in 2 cases and both were reported to have poor compliance (Table 4). Among the post presentation diabetes types, type 1 diabetes mellitus was diagnosed in 13 cases (50%), type 2 diabetes mellitus in 6 cases (23%), impaired glucose tolerance in 1 case (4%) and the rest were not reported (23% - 6/26).

**Table 4:** Order of diagnosis of diabetes mellitus and grave’s disease, past treatment options and compliance

Reference	Order of diagnosis in the past (Diabetes Mellitus and Grave’s disease)	Past anti-thyroid treatment	Anti-thyroid drug compliance	Past diabetes treatment	Diabetes drug compliance	Post presentation diabetes type
[10]	T2D → GD	Methimazole	Poor	Sulfonylurea, thiazolidinedione	N/M	T2D
[11]	GD	Methimazole	Poor	N/M	N/M	T1D
[33]	GD	Methimazole and Lugol solution	Poor	N/M	N/M	IGT
[34]	T2D, GD (Order not mentioned)	Methimazole	Poor	Insulin	Poor	T2D
[35]	Diabetes	N/M	N/M	N/M	N/M	N/M
[35]	Diabetes	N/M	N/M	N/M	N/M	N/M
[35]	Diabetes	N/M	N/M	N/M	N/M	N/M
[35]	Diabetes	N/M	N/M	N/M	N/M	N/M
[35]	Diabetes	N/M	N/M	N/M	N/M	N/M
[36]	N/M	N/M	N/M	N/M	N/M	T1D
[37]	GD	N/M	Poor	N/M	N/M	T1D
[38]	T2D	N/M	N/M	Insulin	N/M	T2D
[39]	N/M	N/M	N/M	N/M	N/M	T1D
[40]	N/M	N/M	N/M	N/M	N/M	T2D
[41]	GD → T1D	Propylthiouracil	Poor	Insulin	N/M	T1D
[42]	T2D	N/M	N/M	N/M	N/M	T2D
[43]	N/M	N/M	N/M	N/M	N/M	T1D
[44]	GD → T1D	Anti-thyroid drug	Poor	Insulin	Poor	T1D
[45]	T1D → GD	Propylthiouracil	Poor	Insulin	N/M	T1D
[46]	T1D	N/M	N/M	Pre-mix insulin	N/M	T1D
[47]	N/M	N/M	N/M	N/M	N/M	N/M
[48]	N/M	N/M	N/M	N/M	N/M	T1D
[48]	N/M	N/M	N/M	N/M	N/M	T1D
[49]	T1D	N/M	N/M	N/M	N/M	T1D
[50]	T2D	N/M	N/M	Glargine-insulin, aspart-insulin, metformin	N/M	T2D
[51]	GD	N/M	Poor	N/M	N/M	T1D

*GD* Graves’ disease, *IGT* Impaired glucose tolerance, *N/M* Not mentioned, *T1D* Type 1 diabetes mellitus, *T2D* Type 2 diabetes mellitus

### Examination findings

On examination, 77% (20/26) had a thyroid goitre. Features of GD and dehydration were reported in 73% (19/26) and 31% (8/26) respectively. Exophthalmos was seen in 8 cases out of which 50% were reported to have bilateral exophthalmos. Also, tremor was observed in 8 patients (31%). Twenty-two (85%) patients reported both systolic and diastolic blood pressure with a mean of 118.8 (SD 25.8) and 62.0 (SD 25.7) mmHg respectively. Moreover, respiratory rate was reported in 9 patients (35%) with a mean of 25.4 (SD 6.4) breaths per minute. Further, Kussmaul breathing was observed in 31% (8/26). Abdominal examination was reported in 35% (9/26), and none had positive findings.

### Treatment modalities and survival

Symptom control of TS was achieved with beta blockers (69% - 18/26) namely, propranolol (78% - 14/18), landiolol, metoprolol, esmolol and practolol (each - 6% - 1/18). Also, digoxin and reserpine were used in one case each. Thyroid hormone synthesis/release was decreased using antithyroid drugs, radioiodine, and surgery. Propylthiouracil (42% - 11/26), methimazole (31% - 8/26) and carbimazole (19% - 5/26) were the thionamides used as anti-thyroid agents. However, the latest 4 cases had methimazole as the anti-thyroid agent. Moreover, iodide treatment was used in 16 patients (62%) and steroids (hydrocortisone - 6, dexamethasone - 3) in 9 patients (35%). Further, thyroidectomy was performed in 15% (4/26) of the reported cases.

Treatment for DKA included insulin with intravenous fluids which were received by all patients. Also, electrolyte replacement was received by 19% (5/26) and sodium bicarbonate by 4% (1/26). Seven patients were reported to have been maintained on insulin after discharge. Thionamide agents were reported to be continued in 4 patients after discharge (Additional file 1). Cardiovascular, renal and gastrointestinal complications were the top three complications (Table 5). Out of the 26 cases, all survived except four (15%). The causes of death were focal bronchopneumonia with thyrotoxic storm [34], sepsis [35], acute respiratory distress syndrome [42] and multi-organ failure [47].

**Table 5:** Disabilities and complications experienced by the patients

System	Frequency out of 26 (%)
Cardiovascular (acute pericarditis, cardiac arrest, Takotsubo cardiomyopathy)	5 (19)
Renal (anuria, perinephric abscess, renal failure, urinary tract infection)	4 (15)
Gastrointestinal tract (hepatopathy, liver failure)	3 (12)
Respiratory (H1N1, influenza B, respiratory failure)	3 (12)
Haematology (disseminated intravascular coagulation, thrombocytopenia)	2 (8)
Metabolic (hyperuricemia, hypoproteinemia)	2 (8)
Neurological/ psychological (encephalopathy, meningism)	2 (8)
General (hyperthermia)	1 (4)
Musculoskeletal (rhabdomyolysis)	1 (4)

## Discussion

The systematic review found only twenty-six cases globally for concurrent presentation of TS and DKA with a mortality rate of 15% which illustrate a rare but, potentially life-threatening condition. The presentation was common among females and in middle age. Female predominance was predictable as both TS and DKA are common among females [3, 7]. Poor drug compliance is a common precipitating factor for both TS and DKA [4, 6]. And, the review too revealed a poor compliance for routine anti-thyroid and anti-diabetic agents among reported cases. The above statement was further supported by elevated HbA_1C_ and T4 levels with a low TSH among all patients with reported measurements. Thus, the rare life-threatening concurrent presentation of TS and DKA is likely with poor drug compliance. Hence, it is essential to maximise patient compliance to anti-thyroid and anti-diabetic agents and monitor the disease control via laboratory tests. Moreover, both TS and DKA have similar predisposing factors [6, 52] therefore, a common trigger could have resulted in triggering them together. Although, most of the cases had a diagnosis of diabetes mellitus preceding that of GD, it was unclear if DKA preceded TS. Nevertheless, Potenza M and colleagues (2009) proposed an initial trigger by excessive thyroid hormone to cause DKA which would subsequently leads to TS [10].

The mean quality assessment score for the selected articles was 19.2 (SD ± 3.6) out of a total of 30 per article. Differences in reporting of clinical presentation, investigation findings and treatment options were noted. These limited the review in producing a comprehensive summary of the simultaneous development of TS and DKA. Prospective studies on concurrent presentation of TS and DKA are not possible due to the rare nature of the condition. However, constant reporting of similar cases is essential for the better understanding of this rare entity.

## Conclusions

The concurrent presentation of TS with DKA is rare but can be life-threatening. Efforts should be made to maximise patient compliance to anti-thyroid and anti-diabetic agents to prevent the above concurrent presentation. Also, monitoring of the disease control should be supported by laboratory investigations. Identification of one entity should alert the treating clinician to look for features of the other entity. Hence, the early detection of the rare presentation could help optimize management with anti-thyroid drugs, insulin and intravenous fluids.

## Additional files

Additional file 1: Datasheet of the review on thyroid storm and diabetic ketoacidosis, 2018. Description of data – This provides the data extracted for the review. (XLSX 42 kb)

Additional file 2: PRISMA 2009 checklist. Description of data – This provides the PRISMA 2009 checklist related to this systematic review. (DOCX 28 kb)

Additional file 3: Summary of scores for items of the CARE checklist. Description of data – This provides the summary of scores for each item of the CARE checklist. (DOCX 20 kb)

## Abbreviations

BWPS: Burch-Wartofsky point scale
CARE: Case reports
DKA: Diabetic ketoacidosis
GD: Graves’ disease
IGT: Impaired glucose tolerance
JTA: Japan thyroid association
N/M: Not mentioned
PICO: Population, intervention, comparison and outcome
PRISMA: Preferred reporting items for systematic survey and meta-analysis
T1D: Type 1 diabetes mellitus
T2D: Type 2 diabetes mellitus
TS: Thyroid storm
