# Supplementary material for: Concurrent presentation of thyroid storm and diabetic ketoacidosis: a systematic review of previously reported cases
Source: BMC Endocr Disord. 2019 May 17;19:49. doi: 10.1186/s12902-019-0374-3 (PMC6525384; doi:10.1186/s12902-019-0374-3)
Supplement: Supplementary file 3 — Summary of scores for items of the CARE checklist. Description of data – This provides the summary of scores for each item of the CARE checklist. (DOCX 20 kb) [file 12902_2019_374_MOESM3_ESM.docx]

| **Additional file 3 - Summary of scores for items of the CARE checklist** | | |
| --- | --- | --- |
| **No** | **Item** | **Frequency out of 26 (%)** |
| 01 | The words “case report” should be in the title along with the area of focus | 5 (19) |
| 02 | 2 to 5 key words that identify areas covered in this case report | 12 (46) |
| 03 | Introduction - What is unique about this case? What does it add to the medical literature? | 13 (50) |
| 04 | The main symptoms of the patient and the important clinical findings | 7 (27) |
| 05 | The main diagnoses, therapeutics interventions, and outcomes | 7 (27) |
| 06 | Conclusion—What are the main “take-away” lessons from this case? | 12 (46) |
| 07 | One or two paragraphs summarizing why this case is unique with references | 15 (58) |
| 08 | De-identified demographic information and other patient specific information | 26 (100) |
| 09 | Main concerns and symptoms of the patient | 26 (100) |
| 10 | Medical, family, and psychosocial history including relevant genetic information (also see timeline) | 26 (100) |
| 11 | Relevant past interventions and their outcomes | 26 (100) |
| 12 | Describe the relevant physical examination (PE) and other significant clinical findings | 26 (100) |
| 13 | Important information from the patient’s history organized as a timeline | 0 (0) |
| 14 | Diagnostic methods (such as PE, laboratory testing, imaging, surveys) | 26 (100) |
| 15 | Diagnostic challenges (such as access, financial, or cultural) | 0 (0) |
| 16 | Diagnostic reasoning including other diagnoses considered | 26 (100) |
| 17 | Prognostic characteristics (such as staging in oncology) where applicable | 0 (0) |
| 18 | Types of intervention (such as pharmacologic, surgical, preventive, self-care) | 25 (96) |
| 19 | Administration of intervention (such as dosage, strength, duration) | 20 (77) |
| 20 | Changes in intervention (with rationale) | 18 (69) |
| 21 | Clinician and patient-assessed outcomes (when appropriate) | 25 (96) |
| 22 | Important follow-up diagnostic and other test results | 12 (46) |
| 23 | Intervention adherence and tolerability (How was this assessed?) | 25 (96) |
| 24 | Adverse and unanticipated events | 19 (73) |
| 25 | Discussion of the strengths and limitations in your approach to this case | 24 (92) |
| 26 | Discussion of the relevant medical literature | 26 (100) |
| 27 | The rationale for conclusions (including assessment of possible causes) | 26 (100) |
| 28 | The primary “take-away” lessons of this case report | 26 (100) |
| 29 | When appropriate the patient should share their perspective on the treatments, they received | 0 (0) |
| 30 | Did the patient give informed consent? Please provide if requested | 1 (4) |
